# Food safety and dietary diversity in African urban cities: evidence from Ghana

**DOI:** 10.1186/s12889-024-18297-0

**Published:** 2024-03-25

**Authors:** Makafui I. Dzudzor, Nicolas Gerber, Felix A. Asante

**Affiliations:** 1 Centre for Development Research (ZEF), University of Bonn, Genscherallee 3, Bonn, D-53113 Germany; 2 Institute for Food and Resource Economics (ILR), University of Bonn, Nussallee 19, Bonn, D-53115 Germany; 3 Institute of Statistical, Social and Economic Research (ISSER), University of Ghana, P.O. Box LG 74, Legon, Accra Ghana

**Keywords:** Food safety, Dietary diversity, Seasonality, Food markets, Urban

## Abstract

**Background:**

Food safety is integral to food security and is increasingly becoming a significant concern in the urban areas of Africa, which are rapidly growing in population. In the case of Ghana, many urban households depend on traditional open-air markets for most of their food needs. However, these urban food markets also depend on domestic food supply chains, which are prone to risks, including poor hygiene and sanitation and weather seasonality. Food safety compliance has associated costs which increase the unit cost of food products. Thus, higher food price is a risk factor to food availability and accessibility—fundamental pillars of food security.

**Method:**

We use food microbial data and food retail data from food market surveys in major cities in Ghana to assess the safety of selected fresh food commodities and how retailers handle the food products they sell. Additionally, based on a two-wave balanced panel household data, we used fixed effects Poisson and Correlated Random Effects (CRE) Probit models to estimate the effect of weather seasonality on the incidence of diarrhoea and urban household dietary diversity score (HDDS). A final sample of 609 households and 565 market respondents participated in the study.

**Results:**

Our findings show that selected food samples tested positive for S*taphylococcus aureus* and *E.coli* and had aflatoxin B1 levels above 5.0 ppb. Additionally, the household incidence of diarrhoea/vomiting, a proxy for food safety status, is higher in the dry season. In the dry season, the household incidence of diarrhoea/vomiting increases on average by a probability of 38% points compared to the rainy season. Regarding HDDS, the average HDDS is 7.3; however, we did not find the effect of seasonality on HDDS to be significant.

**Conclusions:**

Although urban food availability and household dietary diversity are not challenges for many urban households, food safety is a challenge in the major food markets in Ghanaian cities and is associated with weather seasonality. Foods available in traditional open-air markets are not always safe for consumption, undermining households’ food security. Weak enforcement of food safety regulations contributes to the food safety challenges in Ghanaian urban food markets.

**Supplementary Information:**

The online version contains supplementary material available at 10.1186/s12889-024-18297-0.

## Background

The current global food system cannot provide healthy and safe diets inclusively and sustainably [1]. Poor diets account for a significant number of deaths, estimated, for instance, at 1-in-5 deaths in 2017 [2]. Poor diets, including the low intake of whole grains and fruits and the high consumption of sodium, accounted for more than 50% of the deaths related to diet [2]. Additionally, every year, 600 million and 420,000 people fall ill and die, respectively, from eating contaminated food [3]. Food safety has become a public health priority globally [4–6]. Food safety is the assurance that there are no adverse health effects from food prepared and consumed by an individual for an intended purpose [7]. Food safety is intricately and inextricably linked to food security [8]. Food security “exists when all people, at all times, have physical, social and economic access to sufficient, safe and nutritious food that meets their dietary needs and food preferences for an active and healthy life” [9]. Therefore, food safety underpins food security because there can be no food and nutrition security without food safety [10].

Food security has four foundational pillars—food availability, accessibility, utilisation and stability [9, 11]. The recent addition of agency and sustainability to food security pillars highlights the importance of peoples’ rights to determine what they eat and the sustainability of the food system that produces the food people consume [11]. Food safety contributes to food security through food utilisation and stability pillars [11]. However, safe food has associated costs to produce it. The costs of investing in equipment, monitoring and implementing food safety protocols increase the unit cost of production [12, 13], especially in developing countries where firms can be small, and the cost of food safety compliance can be expensive for the firm [14]. Ensuring food safety compliance adds to the food’s unit cost and selling price, thus impacting households’ food availability and accessibility.

Non-compliance with food safety requirements can also lead to food availability and accessibility challenges through increased food waste and disposal [15, 16]. Disposing of unsafe foods contributes to food waste, and unsafe food disposal comes at a cost and thus affects food prices, leading to food availability and accessibility challenges, and it is not environmentally sustainable [16]. For example, to comply with food safety regulations, firms must dispose of unsafe foods, and regulatory agencies discard entire shipments of food products because tested samples do not meet phytosanitary and other regulatory standards [16]. Therefore, food safety compliance not only benefits the consumer in terms of access to safe food consumption and promoting good health, but it also benefits the producer, who can benefit from the growing base of consumers willing to pay premiums for safer foods [17]. Additionally, the producer saves on cost by avoiding punitive fines from regulators, the cost of recalling unwholesome foods, reputational damage, and liabilities [15, 17, 18].

In addition to the food safety challenges facing food systems at various levels, the growing sophistication of consumer demands is increasing the complexity of food systems [1, 4, 5, 19]. The food system’s complexity is compounded by environmental factors like seasonal variations in rainfall and temperature [20]. Seasonality can affect household food consumption decisions and food safety. Seasonality influences household food consumption decisions via the availability of food varieties and diet composition [21–23], food accessibility and price [23, 24] and loss of employment [25]. Seasonality shapes household dietary diversity through demand- and supply-side factors [26]. Seasonality and agricultural production are linked, especially in developing countries and rural areas where most agricultural production is rain-fed [27, 28]. Some foodborne diseases are linked to specific weather and climatic conditions and are prevalent at specific times and seasons of the year [29–31]. For example, the *Salmonella* transmission risk increases with high rainfall [30]. So traditional open-air markets, which are the primary source of food markets for urban households in developing countries [32, 33], can be environments where pathogens can easily find their way into food and water if not hygienically maintained [6, 34].

Depending on the level of market integration with the global food system, seasonality affects the availability and prices of some foods in the market [21, 24]. Households that live in big cities that are well connected to global food systems and have higher income levels have higher dietary diversity [35]. However, the pricing of food products in some of these markets can also be too expensive for the urban poor, thus curtailing their dietary diversity [36, 37]. Developing countries are the most exposed to unsafe foods and food insecurity and the least equipped to manage them. Africa has the highest burden of foodborne diseases per population [3, 6]. Supermarkets are fast becoming regular features of food systems in developing countries [38–40]. Although their impact on diet quality and diversity is mixed [38, 41–43], they are promoted for their improved quality and safety standards along the food supply chain [44].

Therefore, food safety is critical to food security by reducing the risk of foodborne diseases and enhancing food utilisation. However, improved food safety has the potential to increase the cost of food, thus affecting food availability and accessibility, leading to a low household dietary diversity score (HDDS) [45]. HDDS is households’ access to and consumption of different varieties of food groups within a specified period. Therefore, HDDS shows households’ ability to afford a variety of food groups and the availability of the food [46]. Additionally, weather seasonality affects food availability in the market, household dietary diversity, and food safety—manifested in food-related health outcomes like diarrhoea and vomiting. Reviewing much of the existing literature on food security focuses on rural and agricultural households. It concludes that households are more food secure during the harvest season or soon after harvesting [47, 48].

Given the growing share of people living in urban areas and engaged in non-agriculture activities, the study highlights the centrality of food safety in food security considerations and the seasonality of household food safety and HDDS in urban areas in developing countries. Empirical research combining food safety and availability within an urban food security context is limited. Therefore, the paper explores weather seasonality, urban households’ food safety status, and dietary diversity scores. We test the hypothesis that seasonality does not affect HDDS in urban areas with major food markets. We answer the question: What is the effect of seasonality on urban households’ dietary diversity score and the incidence of diarrhoea/vomiting due to food consumed?

## Methods

### Study area

**Fig. 1:**
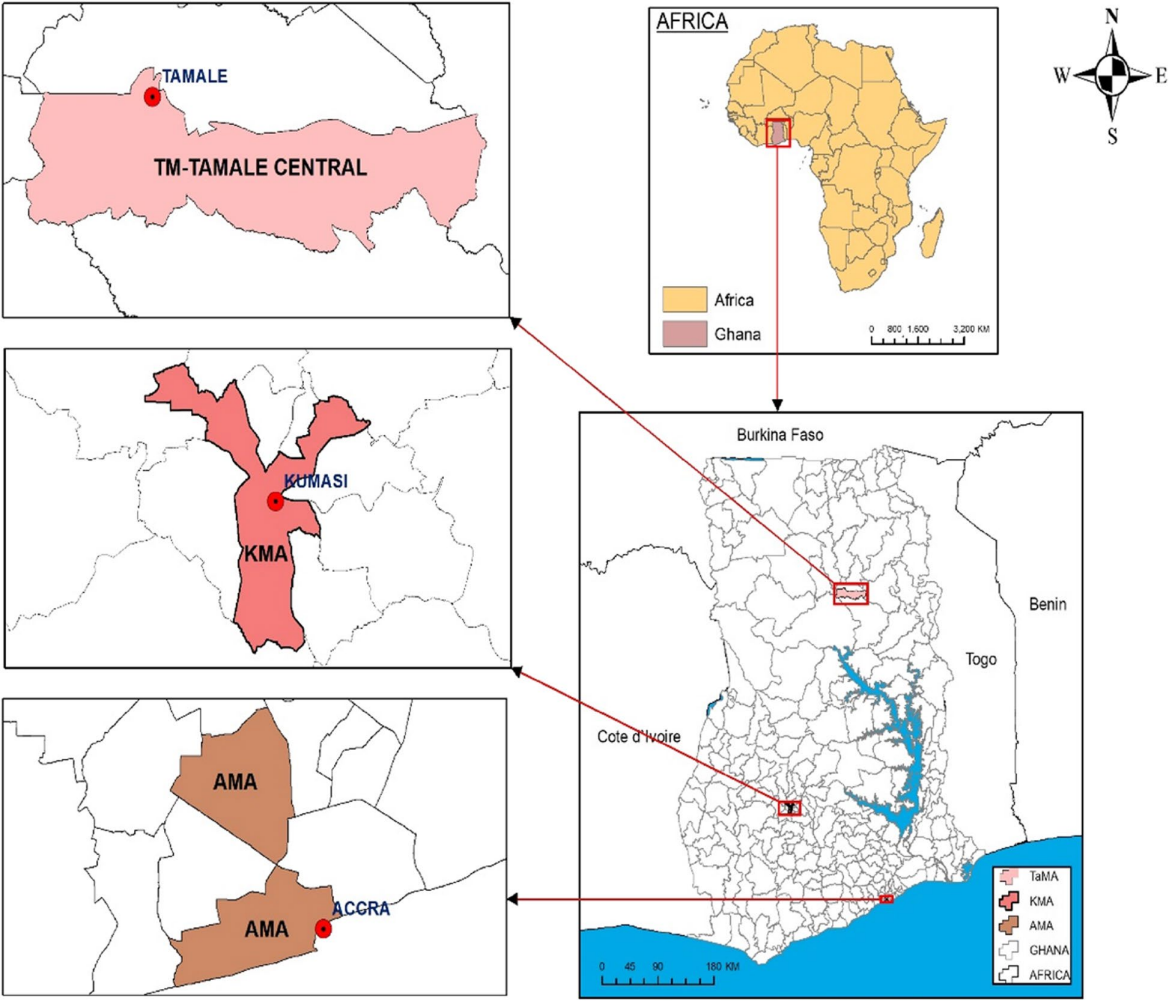
A map of Ghana showing the study sites

The study area is selected cities in Ghana (Fig. 1). The study was conducted in Accra, Kumasi and Tamale Metropolises in Ghana. According to Ghana’s 2010 Population and Housing Census, these cities are the biggest in Ghana’s southern, middle and northern parts. The three cities are cosmopolitan and have major food markets. The different study sites provide different perspectives on the urban food system in Ghana. We presented a detailed description of the three cities in Dzudzor and Gerber [32].

The data used is mainly based on household and market surveys from Ghana. Ghana is a lower-middle-income country in West Africa, with a population of about 31 million [49]. In 2019, the country had a nominal GDP of about US$69 billion [50], with the service sector contributing the largest share of 47.2%, followed by industry with 34.2% and the agriculture sector with a contribution of 18.5% [50]. The agriculture sector remains critical to economic development and serves as the fulcrum for the government’s food and nutrition security agenda. Agriculture remains highly dependent on rainfall. Ghana has different agroecological zones that influence economic and agricultural activities that dominate these regions [51].

As a developing country, urbanisation is on the ascendency. The proportion of the Ghanaian population living in urban areas as of 2021 was 57% [49]. Over the past century (1921–2021), Ghana’s urban population has rapidly increased from about 8% in 1921 [52] to almost 57% of the total population in 2021 [49]. Natural population growth and internal migration increase mainly drive Ghana’s urban population growth [52]. The growth in urbanisation has been geographically disproportional. Almost half (47.8%) of the growth in urbanisation between 2010 and 2021 occurred in just the Greater Accra and Ashanti regions [49].

### Survey data and questionnaire

#### Survey data

This paper uses three data sets: household, market (retail) and fresh food microbial analysis data. The household and market surveys were conducted in all three cities to explore the issues of food safety and nutrition, dietary diversity, and food consumption behaviour of households. Additionally, a food safety analysis was conducted to detect the presence of some foodborne pathogens in food commodities when they arrive and are sold in the Agbogbloshie market in Accra. A total of 66 samples were tested for different foodborne pathogens. A combination of the different data sources provides different perspectives on the urban food system in Ghana. After data cleaning and management, 609 households and 502 retailers’ (market women/men) complete data were analysed (Table 1) across the study sites. A panel was developed with two rounds of household data to account for seasonality. The period between the two rounds of data collection was about six months (November/December 2019 - June/July 2020). Between the two rounds, there was the Covid-19 outbreak that resulted in the partial lockdown of parts of Accra and Kumasi. As a result, households in some Enumeration Areas (EAs) in Accra and Kumasi, with a high concentration of migrants from other regions in Ghana, relocated to their hometowns. These reasons contributed to the fewer respondents in Accra and Kumasi in round 2 of the survey. Tamale had no lockdown, so the same number of respondents were enumerated.

The data collection process was done in line with the farming calendar in Ghana. There are two rainy seasons in the southern and middle parts (Accra and Kumasi) of Ghana: major season (April-June) and minor season (September-October) and in-between the dry season. In northern Ghana, there is a single season. The rainy season is June-August, and the dry season is September-May. The data collection was modelled after these distinct seasons (rainy and dry seasons). The first round of data collection was done in November-December 2019 (dry season). The dry season is the harvest period of most staple foods in Ghana. The second data collection round was conducted from June to July 2020 (rainy season). This is the main cropping season for most crops.

**Table 1:** Data used in the study

Study sites	Round 1	Round 2
***Household survey [number of respondents]***		
Accra	216	175
Kumasi	240	218
Tamale	216	216
** Total**	**672**	**609**
***Market survey [number of respondents]***		
Accra	205	
Kumasi	200	
Tamale	160	
** Total**	**565**	
***Food microbial analysis (Accra only) [number of food samples]***		
Microbial analysis	43	
Aflatoxin B1 analysis	23	
** Total**	**66**	

#### Survey questionnaire

As part of the data collection process, the NOURICITY project team trained enumerators to administer the questionnaires in the local language. During the training, technical terms and concepts were discussed and explained in the local language to ensure all enumerators used the same nomenclature during interviews. After training, we pre-tested the questionnaire. We corrected challenges identified during the pre-testing to develop the final questionnaire we administered.

The household questionnaire administered was structured and had several sections. The sections related to food, safety and nutrition included household demographics (household head, age, sex, marital status, education, household size, employment status, relationship with household head), food security (household food consumption in the last 24 hours and experience any anxiety of not having access to food in any month in the last 12 months), housing characteristics (type of house, number of rooms, type of floor, wall and roofing material, type of toilet facility, type of water source, lighting, and cooking fuel), household assets and income, well-being and food expenditure, food safety knowledge, attitude and practices (KAP) (based on the WHO’s five keys to safer foods) and self-reported health status, access to infrastructure, and household members’ health status related to diet. During the second round, the survey also included questions on the potential effect of Covid-19 on the availability and access to food, changes in the price of food commodities in the market and the employment status of household members.

We utilised the same approach as the household survey to create the final questionnaire for the market survey. The market survey questionnaire consists of various sections. It covered the respondent’s profile (including age, sex, education level, business experience and the type of product sold, such as vegetables, fruits, roots and tubers, grains, pulses, nuts, livestock, meat, dairy products, eggs and miscellaneous items), whether the respondent has migrated from another part of the country, type of business operation, reasons for choosing the business location, working hours and days, level of processed products sold; constraints faced by the business, the respondent’s food safety knowledge, and access to lighting and sanitary facilities.

For the microbial analysis, the team collected information on the lorry tracks when they arrive in the market, the wholesaler whose product it is, the location details of the wholesaler, and the retailers who come to buy from the wholesalers. The enumerators followed the retailer to their selling locations for follow-up data collection. We label all the products by date, name and type of seller before putting them in an ice chest and transporting them to the laboratory for analysis.

### Sampling design

The data used in this study is based on the survey and sampling design employed under the Partnership for Healthy Diets and Nutrition in Urban African Food Systems-Evidence and Strategies (NOURICITY) project. The NOURICITY project was designed and conducted in Ghana, South Africa, and Uganda to investigate the individual and systemic drivers and dynamics of urban food systems in Africa [53]. Regarding research activities in Ghana, we obtained ethical clearance from the Ethics Committee for Humanities, University of Ghana and the Ethics Committee of the Centre for Development Research (ZEF), University of Bonn. In all household and market interviews, we obtained informed consent from respondents.

#### Market survey design

We used a two-stage sampling approach. We used purposive and random sampling approaches in the first and second stages. We used purposive sampling to select three markets in Ghana’s south, middle and northern parts. We selected the markets from Accra, Kumasi and Tamale. The markets selected were the Makola and Agbogbloshie Markets in Accra, Kumasi Central Market in Kumasi and Tamale Central Market in Tamale. The criteria for selecting these cities are as follows: they have major food markets that are hubs for aggregating and redistributing a wide variety of food products to other cities and regions in Ghana and neighbouring countries. Also, these markets play essential roles in the national and regional food systems. Urban households depend directly and indirectly on these markets for their food needs. In addition, retailers in smaller and satellite markets in these cities source many of their products from these major markets for onward sale in communities far from the major markets. The selected markets also provide a reliable outlet for agricultural products from production (rural communities and towns) areas to be sold.

In Accra, we identified the boundaries of the Makola and Agbogbloshie markets and conducted a mapping survey (retailer listing) of the types of food retailers and structures in the markets. Although the selected markets have thousands of actors, there is homogeneity (groups/clusters) in the types of products sold and the structure of the selling outlets. Therefore, we sampled based on products sold and structures in the markets. In the mapping survey, we randomly selected retailers within a particular food commodity cluster for fair geographical distribution—about 1000 retailers were sampled. During sampling, we also sampled retailers among a particular cluster other than the one in which we expected to find them. For example, when a vegetable seller is among cereal (maize) sellers, the vegetable seller is enumerated. After the mapping survey, we randomly sampled about 205 respondents for the market survey, which involved administering a more detailed questionnaire. Based on the experience from Accra, in Kumasi and Tamale markets, we did a recognisance visit to the market to identify the main food clusters based on types of food sold and types of structures (e.g. wholesalers, retailers and immobile hawkers) and their location. After identifying the clusters, we did random walks to enumerate respondents. We sampled 200 and 160 respondents in the Kumasi and Tamale Central markets.

#### Fresh food sample collection technique

We selected four food commodities, picked samples from the Agbogbloshie market in Accra, and performed microbial analysis to determine the presence of some selected foodborne pathogens. We selected tomatoes, cabbage, maize and groundnuts. The microbial analyses performed are total Coliform, *E. coli*, *Staphylococcus*, and *Salmonella* counts, as well as detection tests for *Salmonella spp.* and *Listeria monocytogenes*. The selected foodborne pathogens are some of the most common pathogens that cause food contamination [8]. Further, given the high incidence of aflatoxins in cereals [54], the maize and groundnut samples were tested for Aflatoxin B1 (AFB1) concentrations.

For the fresh food microbial analysis, we focused on traditional open-air markets. We traced the food products from the point when they arrive in the market to the point where it is sold by the final retailers. We traced and collected samples over several days. The food samples were collected at various stages (sampling points) when the food commodities arrived in the market. The first samples were collected when the food trucks arrived at the market (when the trucks were offloading). We assume that samples collected at this stage will capture the conditions of the food commodities from the source of production through the transportation phase to the market. Therefore, we documented the wholesalers who received these goods on the first day. On the second day after the delivery day, we collected the second sample from wholesalers who received the commodities on the first day. The 2-day time span between the first and second samples captures some of the market conditions that affect the food commodities (e.g. environment, sanitation and storage conditions). After another two days, we collected a final food sample from the retailers selling in smaller quantities (most customers buy from these sellers). These final samples are not necessarily from the initial trucks sampled, but they were samples bought by the retailers from similar trucks that delivered the food products on the same day the first samples were taken from the sampled trucks.

Table 2 shows the number of samples collected from the Agbogbloshie market. The testing of the food samples was conducted by and at the Noguchi Memorial Institute for Medical Research (NMIMR), University of Ghana. The NMIMR tested the samples using their “Standard Operating Procedure (SOP) for enumeration and detection of pathogens from food and animals (Bac-047-1.0)” [55]. The SOP outlines the procedures used to analyse the food samples collected. Similarly, they tested the aflatoxin levels in line with Aflatest® High-Performance Liquid Chromatography (HPLC) for corn, raw peanuts and peanut butter [56]. In total, 43 samples were collected and tested for selected food microbes. We also tested 23 samples for Aflatoxin B1. We focused on the common foodborne pathogens linked to sanitation, hygiene and storage. Unfortunately, due to budget constraints, this study could not collect samples from all three major markets surveyed in the study. Therefore, although the total number of food samples collected from the Agbogbloshie market is not nationally representative, it indicates the levels of foodborne pathogens present in food commodities sold in major food markets in Ghana.

**Table 2:** Total food samples tested for selected food pathogens

	Samples tested				
	Tomatoes	Cabbage	Maize	Groundnuts	Total
Microbial analysis	10	11	14	8	43
Aflatoxin B1			13	10	23

*Source*: NOURICITY, 2020

#### Household survey design

We adopted a three-stage sampling approach. In the first stage, we used purposive sampling to select the three biggest cities in Ghana’s south, middle and northern parts based on Ghana’s 2010 Population and Housing Census (PHC). In the second stage, we randomly selected a specified number of Enumeration Areas (EA)—the lowest geographical units demarcated by the Ghana Statistical Service (GSS) for national population census purposes—within each of the three cities. Finally, in the third stage, we randomly selected households from each EA. A detailed description of the design of the NOURCITY household survey is presented in Dzudzor and Gerber [32].

### Key dependent and independent variables

#### Household diarrhoea/vomiting incidence

The primary outcome variable in the study is household incidence of diarrhoea/vomiting and illness from food consumption—the variable is used as a proxy for food safety. The variable is computed as a dummy (1/0) and a count variable. The dummy variable is 1 for households with reported diarrhoea/vomiting and illness from food consumption over the last month and 0 otherwise. The incidence of diarrhoea/vomiting as a count variable is the number of cases of diarrhoea/vomiting and illness from food consumption suffered by household members over the last month. The household incidence of diarrhoea/vomiting and illness from food consumption is based on the self-reported cases of households. The authors acknowledge multiple causes and sources of diarrhoea/vomiting [3, 57]. However, contaminated food and water are the most common sources of diarrhoea [8, 58]. Additionally, there is a positive correlation between food contamination and safety and the incidence of diarrhoea and vomiting [59, 60].

#### Household dietary diversity score (HDDS)

HDDS is another key dependent variable. It is the number of unique food groups the household consumed over a given period. The HDDS is based on a 24-hour recall period to improve the accuracy of the information collected. The HDDS consists of 12 food groups, which are their nutritional values-cereals; roots and tubers; vegetables; fruits; meat, poultry and offal; eggs; fish and seafood; pulses, legumes and nuts; milk and milk products; oil and fats; sugar and honey; and miscellaneous (e.g. condiments, coffee, tea). The HDDS ranges from 0 to 12 for each household, and the average HDDS for the sampled group will be the proportion of the sum of all HDDS to the total number of households sampled. In addition, the HDDS serves as a proxy to measure the socio-economic level of the household, given that a higher HDDS correlates positively with high-quality protein and household income [46].

#### Explanatory and control variables

An explanatory variable in the study is weather seasonality. Weather seasonality is used as a dummy (1/0). Seasonality is assigned a 1 in the dry season (round 1), and 0 is assigned the rainy season (round 2). The next explanatory variable is the average monthly prices of staple crops (maize and tomatoes) in Ghana from 2013 to 2020. The prices are from the weekly food prices collected by ESOKO-Ghana from markets across the country, including the Agbogbloshie, Makola, Kumasi Central and Tamale Central markets. Another explanatory variable is household food safety knowledge. We compute household food safety knowledge as a set of 11 true/false statements on household food safety knowledge. Each household’s total score indicates the level of household food safety knowledge. The questions are from the WHO’s “5 keys to safer foods” [61]. Other control variables include household characteristics like sex, age, education, marital status and employment status of household head; household size; proportion of household members employed; and household wealth status (a proxy for income) computed based on household assets and housing characteristics and amenities.

### Empirical strategy

We are interested in knowing the effect of seasonality on urban households’ dietary diversity and incidence of diarrhoea/vomiting. We use the regression models of the form:

1 $${y}_{it}={\alpha }_{0}+{\alpha }_{1}{S}_{it}+{\alpha }_{2}{\varvec{X}}_{it}+{\alpha }_{3}{\varvec{P}}_{it}+{\in}_{it}$$

where ${y}_{it}$ is the respective outcome variables—HDDS and the incidence of diarrhoea/vomiting. The incidence of diarrhoea/vomiting is both a dummy and a count variable, and HDDS is a count variable. Subscripts $i$ and $t$ denote household observation and time (survey round), respectively. Season ($S$) is a dummy variable. **P** is a vector of prices of staples in Ghana. **X** is a vector of household characteristics. The coefficient${\alpha }_{1}$, measures the effect of seasonality on the outcome variables. We used fixed effect models to control for unobserved time-invariant variables that may influence the outcome variables and other covariates. We use the Poisson fixed effects model to estimate the count outcome variables (HDDS and the number of cases of diarrhoea/vomiting). For the effect of seasonality on HDDS [62–65], the Poisson model can be expressed as:

2 $$Prob\left({Y}_{it}={y}_{it}|{X}_{it}\right)={e}^{{-\lambda }_{it}}{\lambda }_{it}^{{y}_{it}}/{y}_{it}!$$

where $${y}_{it}$$ is the HDDS that varies across households ($$i)$$ and over time $$\left(t\right)$$. We assume the Poisson distribution to have a conditional mean $$\left({\lambda }_{it}\right)$$, which depends on a vector of exogenous variables ($${X}_{it})$$. According to Cameron & Trivedi [65], the conditional mean ($${\lambda }_{it}$$) can be expressed as a log-linear model of the form:

3 $$In {\lambda }_{it}=\beta {X}_{it}+\gamma {Z}_{i}+{\epsilon }_{i}+{\mu }_{t}$$

where ${X}_{it}$ and ${Z}_{i}$ are vectors of time-variant and time-invariant exogenous variables, with $\beta$ and $\gamma$ as the respective vectors of parameters to be estimated, ${\epsilon }_{i}$ represent unobserved household effects, and ${\mu }_{t}$ represents time-specific effects. From Eq. (3), if the unobserved household effects (${\epsilon }_{i}$) are not correlated with any other covariate (${X}_{it}$ and${Z}_{i}$), then we can use random effects panel estimators to achieve unbiased estimates [63, 65]. However, although we assume that weather seasonality is not correlated to other unobserved household characteristics, the unobserved household characteristics may correlate with other covariates in our model. For example, households’ skills, culture and attitudes towards food and health may correlate with their dietary diversity decisions (HDDS) and other covariates like household food safety knowledge, employment and income. For example, higher income correlates with higher HDDS [46], and other household characteristics like education and skills affect employment type and income earnings. Under these conditions, the HDDS will partly depend on the unobserved variables leading to measurement error and endogeneity issues, and the estimated coefficients of HDDS suffer from selection bias [62, 63]. Therefore, we use household fixed effects to control for selection bias and eliminate time-invariant unobserved factors [62, 63]. Additionally, we use household wealth status instead of household income, which is less prone to endogeneity issues in the model [66]. To estimate the effect of seasonality on the incidence of diarrhoea/vomiting (dummy variable), we use Correlated Random Effects (CRE) Probit model. The CRE Probit addresses the incidental parameter problem associated with using Probit fixed effects [67, 68]. The incidental parameter problem arises in panel data analysis when running a non-linear regression (e.g. Logit, Probit) and the time (T) dimension is small (e.g. survey period = 2), and the number of observations (cross-sectional units) is large (N→ ∞). Under such circumstances, only a fixed number of time periods are available to estimate the unobserved heterogeneity parameters for each cross-sectional unit and thus result in inconsistent estimates [67, 69]. The CRE approach accommodates time-constant variables and fixed effects estimates on the time-varying covariates [70]. The CRE estimation can be expressed as follows:

4 $${y}_{it}=\alpha +\beta {x}_{it}+\gamma {\stackrel{-}{x}}_{i}+{r}_{i}+{u}_{it}$$

where ${y}_{it}$ is the incidence of diarrhoea/vomiting status for household $i$ at time $t$, ${x}_{it}$ is the time varying explanatory variables of households, ${\stackrel{-}{x}}_{i}$ is time averages of the time varying explanatory variables, $\beta$ is the fixed effects estimate, (${r}_{i}+{u}_{it}$) is a composite error term, ${r}_{i}$ is the time-constant unobservable variables, and ${u}_{it}$ is the idiosyncratic error term. Adding the time averages (${\stackrel{-}{x}}_{i}$) in the model controls for the correlation between the unobserved effects (${\alpha }_{i}$) and the sequence$\left\{{x}_{it}:t=\text{1,2}\right\}$ [70, 71]. For the robustness check for the effect of seasonality on the number of cases of incidence of diarrhoea/vomiting (count variable), we run CRE Poisson and the Poisson pseudo-maximum likelihood estimator with multiple levels of fixed effects (PPMLHDFE) for our estimation. We use the PPMLHDFE model because of the likelihood of a high number of households that did not experience diarrhoea/vomiting (high number of zeros) in our sample and the non-convergence of the Poisson fixed effects model [72]. All regressions were done using STATA 15 [73].

## Results

### Descriptive statistics

#### Household demographics

Households’ summary statistics are presented in a previous study [32]. In summary, the majority of households are male-headed, the average age of a household head is 47 years, and the average household size is 3.9. Some household characteristics are different across cities. A detailed description of household characteristics is presented in Dzudzor & Gerber [32].

#### Market respondents’ summary statistics

About 89% of the respondents in the market survey are women (Table 3). The average age of respondents is about 44 years. About 23% of the retailers do not have formal education. This percentage is exceptionally high in Tamale, where about 52% of respondents do not have formal education. Retailers in Accra have the highest proportion of migrants from other parts of the country. On average, respondents have been engaged in their retail business for 15 years. The average expenditure per customer among small retailers is GHS14.35. Small retailers form the majority of respondents in the survey. Small retailers are immobile sellers selling food products on the floor, other materials (mats, rigid cardboard and polythene), and table tops. Most small retailers source their fruits and vegetables, cereals, meat and starchy staples locally. They source most of their commodities from within the market from distributors/transporters who bring the products directly to the market. Respondents can access waste disposal bins, toilet facilities and running water in the various markets. However, only 24% of respondents in the Tamale market have access to running water. In addition, market supervision by health and sanitation officers of the local assembly is low. About 42% of respondents have never received any form of visitation from any sanitation officer since they started operating their business at their current location.

**Table 3:** Market respondents’ summary statistics

Variable	Accra	Kumasi	Tamale	Total
% female respondents	92.68	89.00	82.50	88.50
Age(mean)	44.59 (*10.72*)	44.52 (*12.27*)	41.31 (*13.38*)	43.64 (*12.14*)
% respondents with no education	12.68	11.50	51.88	23.36
% respondents who migrated to current location to do business	34.63	4.00	6.88	15.93
Average length of doing business (years)	13.61 (*9.64*)	15.60 (*11.19*)	14.46 (*10.31*)	14.55 (*10.42*)
***Sample size of all retailers (N)***	***205***	***200***	***160***	***565***
Average purchase per customer (small retailers) [GHS]	15.28 (*16.58*)	13.51 (*14.72*)	14.28 (*14.22*)	14.35 (*15.26*)
***Sample size of small retailers (n)***	***121***	***127***	***90***	***338***
** Do you have access to**: (%)				
Waste disposal	78.54	66.50	63.75	70.09
Toilet facilities	90.73	80.50	88.75	86.55
Running water	80.00	61.50	23.75	57.52
** Visit from sanitation officers/inspectors (%)**				
Never	29.76	47.00	51.25	41.95
Annually	32.20	13.00	16.25	20.88
Monthly	18.05	16.00	6.25	13.98
Weekly	6.83	6.00	12.50	8.14
Quarterly	7.32	8.00	5.63	7.08
Daily	1.95	9.00	1.88	4.42
Bi-annually	3.41	0.50	3.13	2.30
Fortnightly	0.49	0.50	3.13	1.24
*N*	*205*	*200*	*160*	*565*
**Source of primary product sold (%)**				
Within the market	42.93	65.50	29.38	47.08
Other sellers within the region	21.46	12.00	22.50	13.81
Other sellers outside the region	8.29	7.00	12.50	13.63
Other sellers within this community	14.63	7.50	16.25	12.57
Own production	10.24	7.50	14.38	10.44
Outside the country	2.44	0.50	3.13	1.95
Others	0.00	0.00	1.88	0.53
*N*	*205*	*200*	*160*	*565*
**Most important cost constrain (%)**				
Transportation	44.39	48.00	32.50	42.30
Staffing (wages)	1.95	0.00	0.00	0.71
Storage	6.34	10.50	10.00	8.85
Spoilage	12.68	17.00	18.13	15.75
Debts	9.27	6.50	14.37	9.73
Rent of trading spot	11.71	13.50	11.25	12.21
Rent of living space	0.98	0.00	1.25	0.71
Electricity	11.71	3.00	3.75	6.37
Other	0.98	1.50	8.75	3.36
*N*	*205*	*200*	*160*	*565*

Note: Standard deviation in parenthesis for averages

### Food prices and retailers’ food safety knowledge

#### Food retailers’ food safety knowledge

More than 55% of retailers know at least one safety measure about their products (Table 4). However, there are variations in the proportion of sellers aware of safety measures in Accra, Kumasi and Tamale. Higher proportions of sellers of meat products, pulses and vegetables are aware of the food safety issues related to the products they sell. Further inquiry into these food safety issues shows that they are mostly related to food preservation and how to maintain a longer shelf life of the products. This indicates that retailers are mainly driven by profit motives and not necessarily safety concerns. For example, most tomato and yam sellers indicated that “heat” (high temperatures) is unsuitable for their products, so they ensure they store them in cool and ventilated places. On the other hand, sellers of cereals and dry pulses were concerned about moisture. They mentioned that a moist environment makes moulds develop on their products and decreases their shelf life, so they have to wrap their products and keep them on shelves to avoid contact with moisture and dust.

**Table 4:** Awareness (self-reported) of food safety issues linked to main food product sold

	Accra		Kumasi		Tamale		Total		*p*-value
	%	*N*	%	*N*	%	*N*	%	*N*	
% of …retailers									
Vegetables	45.8	48	56.4	55	78.2	37	58.57	140	0.0090***
Fruits	33.3	3	47.1	17	71.4	7	51.85	27	0.4708
Roots/tubers/plantain	28.6	14	51.9	27	88.2	17	56.90	58	0.0021***
Dry grains	62.5	16	47.4	19	57.9	19	55.56	54	0.6608
Pulses	50.0	6	28.6	14	100.0	12	59.38	32	0.0003***
Starchy staples	55.6	18	20.0	10	81.8	11	53.85	39	0.0152**
Meat (fresh meat)	37.5	8	70.0	10	77.8	9	62.96	27	0.2110
Total	46.90	113	49.34	152	78.57	112	57.29	377	0.0000***

ANOVA conducted across cities. *** *p*<0.01, ** *p*<0.05, * *p*<0.1

#### Seasonality and price of food commodities

**Fig. 2:**
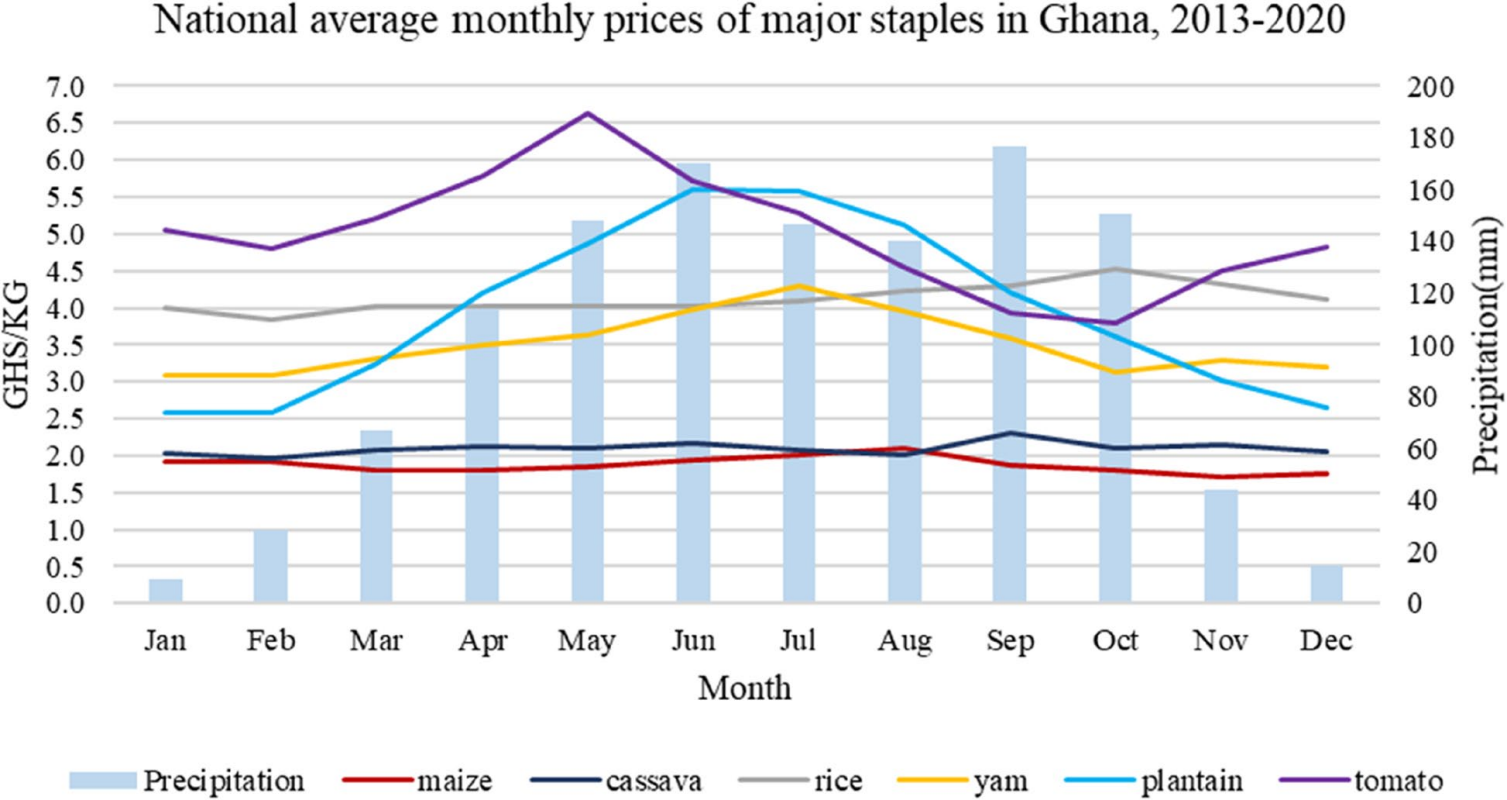
National average monthly prices of major food staples in Ghana, 2013–2020. Source: Authors’ construction, 2021 based on data from ESOKO-Ghana [77] and World Bank [78]

Food prices show some seasonal trends (Fig. 2). Maize, cassava and rice prices show lower seasonal fluctuations than yam, plantain and tomatoes. Food price volatility is a significant issue in Ghana [74]. Critical causes include high dependence on rain-fed agriculture, poor storage facilities and lack of value addition and processing. Therefore, producers quickly flood the market with their products when they harvest to minimise spoilage (e.g. perishables like tomatoes), resulting in oversupply and a sharp price fall. Also, the food supply is low in the lean season, resulting in high prices. Additionally, there is a growing demand for staples like maize for alternative uses besides food. The poultry industry’s use of maize as feed [75] puts pressure on the price of maize in the market. The effect of these price changes on urban households who buy most of their food commodities from the market is likely higher than their rural counterparts. Therefore, weather seasonality affects food prices, leading to potential household food accessibility and dietary diversity challenges [76].

### Food safety and foodborne microbial analysis

From the microbial analysis (Table 5), no Salmonella spp. was enumerated from cabbage, tomatoes, maize or groundnut samples. Similarly, no E. coli was enumerated from cabbage, tomatoes and groundnut samples except for maize. One maize sample from a wholesaler had an *E. coli* level of 1.71 log cfu/25g. *Staphylococcus aureus* was seen in the sampled cabbage from one of the trucks and a groundnut wholesaler. *Listeria spp.* and *Salmonella spp.* were not detected in any of the 43 samples tested. However, *Enterococcus faecalis* was observed in one maize sample from a wholesaler, a tomato wholesaler and retailer, and groundnuts from a truck.

The aflatoxin analysis found that both maize and groundnut samples contained high levels of aflatoxins (AFB1). The European Union (EU) has set a limit of 5.0 ppb for AFB1 in maize meant for human consumption. In comparison, groundnuts have a limit of 2.0 ppb for direct consumption and 8.0 ppb for those undergoing sorting and other physical treatment [79]. According to Ghana’s National Policy for Aflatoxin Control in Food and Feed (NPACFF), Ghana has not set aflatoxin-acceptable limits for all food items [54]. However, it has set the limit for groundnuts to be 5.0 ppb. The results show that only one out of the thirteen maize samples had AFB1 levels below 5.0 ppb—maize sample from a truck had AFB1 value of 4.9 ppb—while all groundnut samples exceeded the limits of 2.0 ppb and 8.0 ppb, except for one sample from a groundnut retailer with an AFB1 value of 6.8 ppb.

If these food products are used for food and animal feed, the US Food and Drugs Administration (FDA-US) has set a permissible limit of 20.0 ppb for total aflatoxin (AFB1, AFB2, G1, G2) [80, 81]. However, some samples had only AFB1 levels exceeding 20.0 ppb. Three of the thirteen maize samples (23.1% of total samples) had AFB1 concentrations above the permissible limit of 20.0 ppb. One of the wholesalers who processed maize into corn dough^1^ had an AFB1 concentration level of 23.1 ppb, while two of the three maize retail samples had AFB1 concentrations of 25.3 ppb (corn dough) and 33.4 ppb (maize grains). Similarly, one out of ten groundnut samples had AFB1 above 20.0 ppb—a sample from one of the groundnut trucks had an AFB1 level of 27.3 ppb. The other raw, roasted, and paste groundnut samples had AFB1 levels lower than 20.0 ppb.

**Table 5:** Presence of selected foodborne pathogens in selected purchased food commodities

**A**		**Microbial levels**			
**Commodity**	**Sampling Point**	**No. of samples tested**	***E. coli ***(log cfu/25 g)	***Staphylococcus *spp**. (log cfu/25 g)	***Salmonella *spp**. (log cfu/25 g)
Cabbage	Trucks	3	0.00	**3.99**	0.00
	Wholesalers	4	0.00	0.00	0.00
	Retailers	4	0.00	0.00	0.00
Tomatoes	Trucks	3	0.00	0.00	0.00
	Wholesalers	3	0.00	0.00	0.00
	Retailers	4	0.00	0.00	0.00
Maize	Trucks	3	0.00	0.00	0.00
	Wholesalers	7	**1.71**	0.00	0.00
	Retailers	3	0.00	0.00	0.00
Groundnuts	Trucks	3	0.00	0.00	0.00
	Wholesalers	1	0.00	**3.00**	0.00
	Retailers	4	0.00	0.00	0.00
**B**		**Aflatoxin B1 contamination**			
**Commodity**	**Sampling Point**	**No. of samples tested**	**No. of samples with AFB1 permissible limits**		
			**(> 5.0 ppb)**^**a**^	**(> 8.0 ppb)**^**a**^	**(> 20.0 ppb)**^**b**^
Maize	Trucks	4	3		0
	Wholesalers	6	6		1
	Retailers	3	3		2
**Total**		**13**	**12**		**3**
Groundnuts	Trucks	3	3	3	1
	Wholesalers	2	2	2	0
	Retailers	5	5	4	0
**Total**		**10**	**10**	**9**	**1**

*Source*: Summary based on results of samples tested at NMIMR

^a^EU Commission Regulation (EU) 2023/915 of 25 April 2023 on maximum levels for certain contaminants in food and repealing Regulation (EC) No 1881/2006 has set the permissible limit at less than 5.0 ppb for maize, less than 2.0 ppb for groundnut for direct consumption and less than 8.0 ppb for groundnut subjected to sorting and other physical treatment

^b^US Food and Drugs Administration set the total aflatoxin action limit to food and feed at 20.0 ppb

### Household dietary diversity and food related health status

#### Household dietary diversity score (HDDS)

**Fig. 3:**
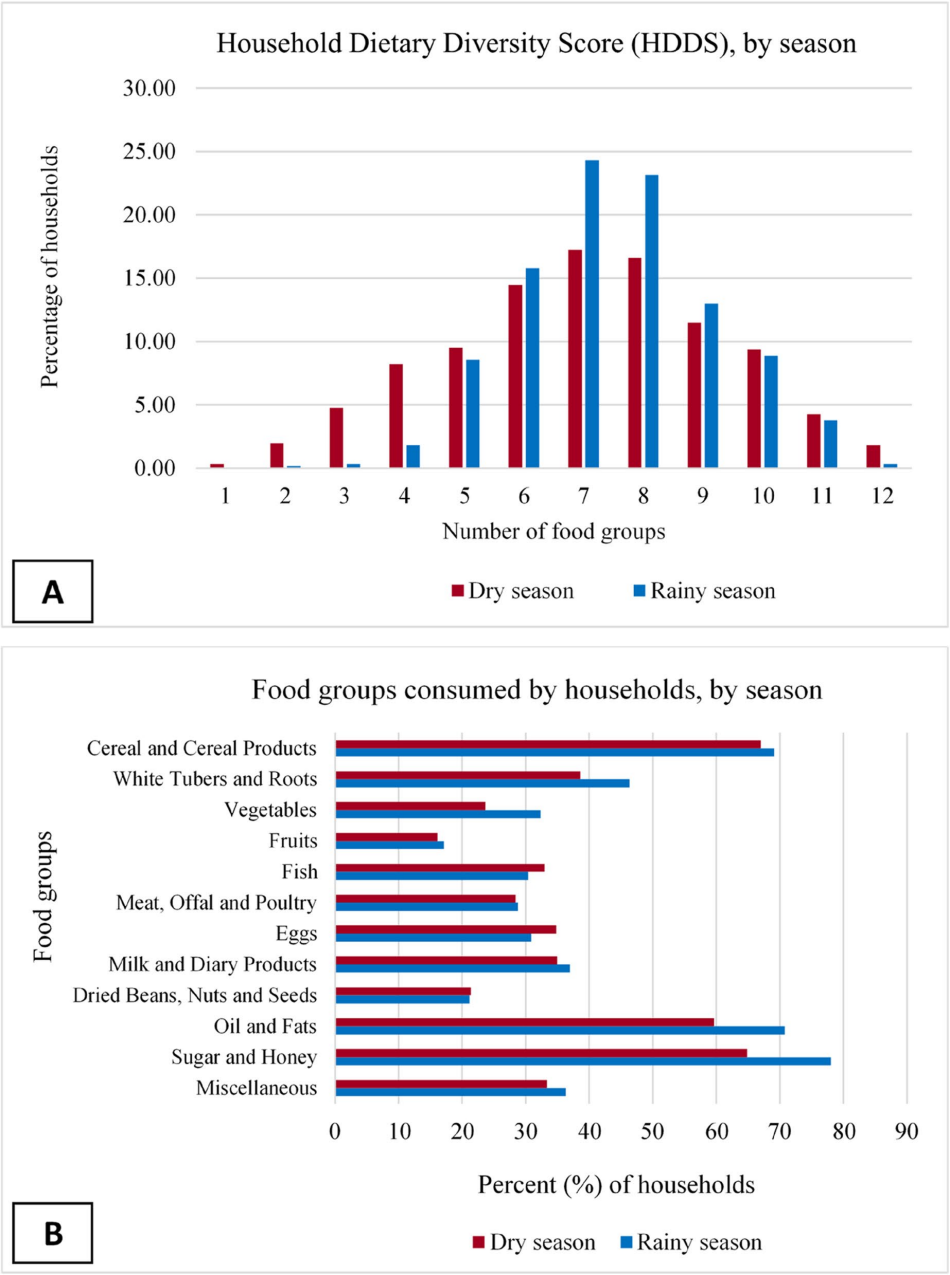
**A **Household Dietary Diversity Score; **B **Food groups consumed by households over the past 24 h by season

HDDS is presented in Fig. 3; Table 6. Figure 3a presents the percentage of households consuming different numbers of food groups in the dry and rainy seasons. Most households consumed between 6 and 9 food groups during the two periods. However, HDDS was higher in the rainy season (7.5) than in the dry season (7.0) (Table 6). Accra and Kumasi have similar and higher HDDS over the two periods compared to households in Tamale. For the specific food groups consumed (Fig. 3b), cereal and cereal products, oils and fats, and sugar and honey products were consumed by more than 50% of households in both seasons. Most households also consumed white tubers and roots. The share of households that consumed oil and fats, and sugar and honey products increased by more than 10% in the rainy season. Similarly, the share of households that consumed vegetables increased by about 9% in the rainy season. The share of households that consumed fruits, meat, offal and poultry, and dried beans, nuts and seeds were broadly similar between seasons.

**Table 6:** HDDS by season and cities

Cities	Mean scores			Diff. across rounds ( 2–1)
	Dry season^1^	Rainy season^2^	Total	
**HDDS**				
Accra metropolis	7.091	7.577	7.334	0.486**
Kumasi metropolis	7.151	7.638	7.394	0.487**
Tamale metropolis	6.907	7.356	7.132	0.449**
Overall (*N* = 609)	7.048	7.521	7.284	0.473***

Significance level: *** *p*<0.01, ** *p*<0.05, * *p*<0.1

Superscript numbers indicate how the difference between dry and rainy seasons are computed (Diff. across rounds (2-1))

#### Household health and diet

Table 7 presents households’ illnesses related to food contamination. More households suffered diarrhoea/vomiting in the dry season than in the rainy season. About 9 and 8% of households suffered from diarrhoea or vomiting in the dry and rainy seasons, although this difference is not significant. More households also suffer illnesses from consuming food away from home than home-cooked food. On average, 10% of households suffered from illnesses related to food consumed away from home compared to about 3% of households who suffered from illnesses related to food consumed at home. The difference in illness attributed to food consumed away from home and food consumed at home is statistically significant, indicating a lesser food safety status of food away from home.

**Table 7:** Household health and diet related illness

	Dry season^1^	Rainy season^2^	Total	Difference (2 − 1)
Suffered diarrhoea or vomiting (%)	9.195	7.882	8.539	-1.313
Illness related to food consumed away from home (%)^a^	10.345	10.181	10.263	-0.164
Illness related to food consumed at home (%)^b^	2.299	2.956	2.627	0.657
Difference between (a-b)	8.046***	7.225***	7.635***	
Total number of households (N)	609	609	1218	

*** *p* < 0.01, ** *p* < 0.05, * *p* < 0.1; t-test of diff. between illness resulting from food consumed away from home and food cooked at home are statistically significant

The superscripts indicate how the difference (2-1) or (a-b) are computed

### Effect of seasonality on household incidence of diarrhoea/vomiting

Table 8 presents the results of Correlated Random Effects (CRE) Probit estimations. From the analysis, weather seasonality affects the incidence of diarrhoea/vomiting among urban households when controlling for household fixed effects. Columns 1 and 2 present the regression results with and without households’ self-reported effect of Covid-19 on food prices, respectively. The results show that the incidence of diarrhoea/vomiting in sampled urban households is higher in the dry season compared to the rainy season. The results show that all things equal, in the dry season, the incidence of diarrhoea/vomiting increases by a probability of 0.38 compared to the rainy season. The literature on diarrhoea infections in Ghana shows that diarrhoea is seasonal and that children are the most vulnerable.

The results also show that the incidence of diarrhoea/vomiting varies across household heads’ educational levels. In households where the household head has a primary education, diarrhoea/vomiting decreases by a probability of 0.158 compared to households with no formal education. Furthermore, the results also show that the price of maize is positively associated with the incidence of diarrhoea/vomiting. All things equal, a one-unit change in the price of maize increases the probability of the incidence of diarrhoea/vomiting by 1.6. The incidence of diarrhoea/vomiting is higher in households that said Covid-19 affected the price of staple foods than those who did not. The contrary was observed for households who said Covid-19 affected the prices of vegetables.

We checked the robustness of our results on the association between weather seasonality and the incidence of diarrhoea/vomiting (number of cases of diarrhoea/vomiting) (Supplementary Table 1, Additional file 1). We run CRE Poission and, due to the high number of households that did not experience diarrhoea/vomiting (high number of zeros), we applied the Poisson pseudo-maximum likelihood regressions (PPML) with multi-way fixed effects [72], which can control for the high number of zeros in the estimation. The CRE Poisson and PPMLHDFE results show a positive and statistically significant difference in the incidence of diarrhoea/vomiting between seasons: the number of cases of diarrhoea/vomiting is higher in the dry season than in the rainy season. This is consistent with the results of the CRE Probit estimation (Table 8). Comparing the magnitude of the coefficients of CRE Poisson and PPMLHDFE show that PPMLHDFE has a higher magnitude for the association between weather seasonality and the incidence of diarrhoea/vomiting. The difference in the magnitude of the coefficients can be attributed to the ability of the PPMLHDFE to handle the high number of zeros in the model.

**Table 8:** Effect of seasonality on households’ incidence of diarrhoea/vomiting

Variables	1		2	
	Coef.	AME	Coef.	AME
Season (Dry)	2.499**	0.378***	2.453**	0.383***
	(1.061)	(0.134)	(1.034)	(0.134)
Characteristics of household				
Age of household head	0.005	0.001	0.019	0.003
	(0.053)	(0.007)	(0.054)	(0.007)
Sex of household head (male)	-0.838	-0.126	-1.271	-0.209
	(0.823)	(0.141)	(0.852)	(0.164)
Household size	0.036	0.005	0.016	0.002
	(0.164)	(0.022)	(0.166)	(0.023)
Education of household head^*^				
Primary	-5.608***	-0.158***	-4.991**	-0.158***
	(2.021)	(0.008)	(1.957)	(0.008)
Secondary	-1.872	-0.330	-0.966	-0.155
	(1.728)	(0.329)	(1.600)	(0.303)
Tertiary	0.199	0.029	0.991	0.205
	(2.526)	(0.406)	(2.443)	(0.671)
*Household wealth status*				
Lower-middle	0.017	0.002	-0.078	-0.010
	(0.302)	(0.041)	(0.298)	(0.039)
Middle	-0.167	-0.021	-0.204	-0.026
	(0.349)	(0.042)	(0.346)	(0.041)
Upper-middle	-0.425	-0.049	-0.445	-0.051
	(0.382)	(0.037)	(0.386)	(0.037)
Upper	0.125	0.018	0.152	0.022
	(0.466)	(0.068)	(0.466)	(0.071)
Household food safety knowledge	0.007	0.001	0.007	0.001
	(0.008)	(0.001)	(0.008)	(0.001)
*Marital status of household head*				
Single	-0.321	-0.038	0.235	0.035
	(1.438)	(0.148)	(1.353)	(0.225)
Monogamous	0.516	0.068	0.591	0.080
	(0.885)	(0.118)	(0.849)	(0.116)
Polygamous	3.570	0.838***	1.825	0.486
	(3.146)	(0.250)	(3.068)	(0.999)
Price of maize^a^	11.977**	1.612**	12.638**	1.726**
	(5.464)	(0.722)	(5.437)	(0.733)
Price of tomatoes^a^	0.173	0.023	0.137	0.019
	(0.192)	(0.026)	(0.184)	(0.025)
*Employment status*				
Employment status of household head	0.122	0.016	0.193	0.024
	(0.344)	(0.042)	(0.343)	(0.040)
Percent of household members employed	0.009	0.001	0.008	0.001
	(0.006)	(0.001)	(0.006)	(0.001)
*Self-reported covid-19 effect*				
Affected price of staple foods	0.613**	0.103**		
	(0.262)	(0.052)		
Affected price of vegetables	-0.772**	-0.072***		
	(0.356)	(0.021)		
Constant	-1.862*		-1.720*	
	(1.005)		(1.000)	
Time varying averaged regressors	Yes	Yes	Yes	Yes
Self-reported covid-19 effect	Yes	Yes	No	No
Number of observations	1212	1212	1212	1212
Number of unique households	606		606	

Robust standard errors in parentheses. *** *p* < 0.01, ** *p* < 0.05, * *p* < 0.1

Coef.-CRE Probit coefficients; AME-Average Marginal Effects

*Reference base for educational level is “No formal education”

^a^Real price of maize and tomatoes are computed based on ESOKO-Ghana December and June price averages from 2013 to 2020

### Effect of seasonality on HDDS

Table 9 shows the effect of weather seasonality on HDDS. We present the results of a Poisson fixed effects estimation for HDDS. We did not compute marginal effects for the Poisson estimation because the coefficients can be interpreted as semi-elasticities [65].

The results indicate that weather seasonality does not have a statistically significant effect on urban households’ HDDS when household fixed effects are controlled. Other regression results show that large household sizes positively affect HDDS. A unit increase in household size is associated with a 3.4% increase in HDDS. Furthermore, the household wealth index has a positive effect on HDDS. Wealthier households eat more diversified foods. A unit increase in wealth index would result in 3.6% increase in HDDS. It implies that a marginal increase in households’ wealth status increases the number of food groups households consume by 3.6%. Household knowledge of food safety is positively associated with HDDS. A unit increase in household food safety knowledge will translate into a 0.4% increase in HDDS. This result may be attributable to the general positive effect of increased knowledge of food safety. However, there was no statistically significant difference in HDDS of households who attributed price changes in fruit and vegetables and staple foods to Covid-19 and those who did not.

We assessed the robustness of our results using different estimation approaches. We ran pooled Poisson and CRE Poisson estimations for HDDS (Supplementary Table 2, Additional file 1). The pooled Poisson results show that seasonality affects HDDS. HDDS is lower in the dry season compared to the rainy season. All other things equal, the coefficient indicates that HDDS decreases by 5.4% in the dry season compared to the rainy season. When using CRE Poisson, HDDS is lower in the dry season; however, the difference is not statistically significant. Therefore, the Poisson fixed effects and CRE Poisson results are consistent.

**Table 9:** Effect of seasonality on HDDS using Poisson fixed effects

Variables	HDDS
Season (Dry)	-0.025
	(0.120)
Characteristics of household	
Age of household head	0.005
	(0.009)
Sex of household head (Male)	0.237
	(0.146)
Household size	0.033**
	(0.017)
Education of household head^*^	
Primary	0.163
	(0.154)
Secondary	0.088
	(0.178)
Tertiary	-0.318
	(0.363)
Household wealth index	0.035***
	(0.009)
Household food safety knowledge	0.004**
	(0.001)
*Marital status of household head*	
Single	-0.929***
	(0.277)
Monogamous	-0.459***
	(0.098)
Polygamous	-0.676*
	(0.372)
Price of maize^+^	0.136
	(0.641)
Price of tomatoes^a^	0.016
	(0.023)
*Employment status*	
Employment status of household head	-0.010
	(0.043)
Percent of household members employed	0.001*
	(0.001)
*Self-reported covid-19 effect*	
Affected price of staple foods	-0.031
	(0.030)
Affected price of vegetables	0.027
	(0.034)
Constant	
Observations	1,212
R-squared	
Number of unique respondents	606

Robust standard errors in parentheses. *** *p* < 0.01, ** *p* < 0.05, * *p* < 0.1

* Reference base for educational level is “No formal education”

+Real price of maize and tomatoes are computed based on ESOKO-Ghana December and June price averages from 2013 to 2020

## Discussion

### Food safety in cities in Ghana

Our market and microbial food analysis results show that food safety is a challenge in major food markets in cities in Ghana. Foodborne pathogens like *Staphylococcus aureus, Enterococcus faecalis, E. coli* and aflatoxins (AFB1) were present in selected food commodities at different sampling points in the market. Some of the tested foodborne pathogens are present before the food commodities get to the market, and the market environment also potentially introduces or spreads other pathogens. The presence of *Enterococcus faecalis* in some samples is indicative of faecal contamination. The evidence of faecal matter contamination in some food samples raises concerns about the safety and wholesomeness of water used for cultivating vegetables, the personal hygiene of the transporters and the vehicles in which these products are transported and the hygiene and sanitation conditions that pertain in the market. *E. coli* and *Enterococcus faecalis* are linked to cholera and diarrhoea [57]. All the sacks of groundnut had high levels of aflatoxin contamination before arrival in the market; this indicates that aflatoxin contamination occurs during the production and transportation stages of the supply chain. Also, the high aflatoxin levels in maize at different sampling points show that aflatoxin contamination can occur at any stage of the supply chain if the products are not correctly handled. This is because samples taken from all three maize trucks had AFB1 concentrations less than 20.0ppb, but as the products were stored with other bags of maize and processed into corn dough, higher aflatoxin contamination occurred. Mixing different sacks of maize from different sources during storage can lead to contaminated maize, affecting good maize that may have arrived from the farm.

From the self-reported responses of retailers, food retailers have limited food safety knowledge. Although about 57% of sampled food retailers have some food safety knowledge of the commodities sold, this knowledge is mainly linked to food preservation and how to extend the shelf life of their commodities. The implication is that reducing associated costs is important to food retailers relative to other food safety considerations. Most food retailers sell the same types of commodities throughout the year. Thus, they know how to handle their products under different environmental conditions to optimise their profits. Selling the same types of food commodities throughout the year also indicates all-year food availability. However, because of seasonal price changes, urban households may suffer from food accessibility (affordability), adversely affecting the food security status of urban households, especially the vulnerable. Households whose food security status is threatened by higher prices are likely to shift to the consumption of lower quality foods, including the consumption of foods low in dietary diversity and unsafe foods, which increases the risk of food poisoning, disease and malnutrition [82, 83].

Within the household, weather seasonality affects households’ food-related diarrhoea/vomiting infections (food safety). Households in the dry season recorded more cases of diarrhoea/vomiting than in the rainy season. Previous studies in Ghana [84–86] are in tandem with our results that the incidence of diarrhoea is higher in the dry season and also influenced by the wealth status of households and source of food purchases [59]. The seasonal incidence of diarrhoea is also consistent with the literature that shows that the quantity of water supply and water availability to households strongly affects their WASH behaviour and health outcomes [87, 88]. So, households suffer from food-related diarrhoea/vomiting in the dry season, potentially because inadequate or inconsistent access to improved water heightens the risks of noncompliance with WASH behaviour [89]. So, a constant safe water supply improves households’ WASH behaviour and health outcomes. Our findings are inconsistent with studies by Anyorikeya et al. [90] and Asamoah et al. [91], who found the incidence of diarrhoea to be higher during the rainy season in their study areas.

The incidence of diarrhoea/vomiting is lower among household heads with a higher formal education than those with no formal education. Kumi-Kyereme and Amo-Adjei [92] found that in Ghana, children in households with higher wealth status and mothers with higher formal education had lesser odds of suffering from diarrhoea. Generally, socioeconomic disparities significantly influence households’ access to resources, including healthcare. Therefore, as households’ socioeconomic status (including wealth status and education) improves, incidences of diarrhoea are likely to decline [93, 94]. Similarly, increases in the price of staple foods like maize imply that food-insecure households adopt coping strategies that may compromise their food quality. They shift to consuming food of lesser quality leading to increased risk of disease and essential nutrition deficiency [79].

Households attributed suffering more food contamination from consuming food outside the home than from eating home-cooked food. Also, households with more employed people outside the house tend to eat food cooked outside the home. There are hygiene and food safety concerns in Ghana regarding food vendors and eateries. Major food contamination incidences have occurred at both high-end restaurants and street food vendors, with some resulting in deaths [95, 96]. In addition, quality food outside the home is expensive [97]. So, consumers may rely on the trustworthiness and reputation of the food outlet [98] to continue patronising their food so long as they do not fall sick. Thus, consumers face a higher risk of food contamination in Ghana when patronising food outside the home [59].

### Food availability and HDDS in cities in Ghana

Our household results also show that the proportion of households that consume different food groups varies between the dry and rainy seasons. The proportion of households that consumed animal-based protein foods (egg and fish) declined in the rainy season, while meat, offal and poultry increased marginally. The greater availability and affordability of green leafy vegetables during the rainy season increased household consumption, making them a healthy option to add to food dishes. However, the increase in the share of households consuming oil and fats, and sugar and honey products are not necessarily healthy diet options. Consequently, avoiding and eliminating industrially-produced *trans-fatty* acids in the food system should be promoted [99]. In addition, balanced consumption of sugar and healthier oils, like olive, canola, coconut, and avocado, should be promoted and made affordable because oil and fats, and sugar and honey are common ingredients in most dishes in developing countries.

When controlling for household fixed effects, we did not observe a statistically significant effect of weather seasonality on HDDS in urban Ghana. This may be because urban households, especially in major cities, purchase most of their food from traditional open-air markets [32, 33]. In addition, major urban markets have more diversified food supply sources (Table 3), so food availability is less of a challenge in urban markets. The different food markets and the diversified food supply sources curtail the effect of weather seasonality on HDDS. However, weather seasonality can affect household food consumption through the prices of food commodities. Kaminski et al. [76] showed that seasonal food price changes are inversely correlated with household food consumption. From our analysis, the price of maize and tomatoes has a minimal or no effect on HDDS when controlling for household fixed effects. This may be because HDDS counts the number of food groups a household consumes and does not measure the quantity and quality of food consumed. Thus, the quantity of food consumed by households from different food groups may change between seasons, but the dietary diversity score recorded may remain the same. However, wealthier households eat more diversified foods and spend more on food per capita, a potential indication of healthier food choices. Wealthier households can spend more on food per capita and consume more diversified foods within food groups and more food groups [46, 100].

Also, household size is positively associated with HDDS. The finding is corroborated by Thorne-Lyman et al. [45], who found that household size positively correlates to dietary diversity and negatively correlates to per capita monthly total food expenditure. Household size can positively affect dietary diversity because, with many household members and their varied ages, the household is likely to have higher income and consume food with high dietary diversity to meet members’ nutritional needs [101, 102]. Conversely, household size can negatively affect household dietary diversity. Due to large family sizes, poor households may not be able to spend more on adequate nutritious food and thus reduce their diet quality and diversity to meet the hunger needs of all household members [103, 104].

### Limitations of study

Even with our findings, the study may be constrained by the definition of some variables. An index to measure food safety based on more indicators is more desirable than the use of the incidence of diarrhoea as a proxy for food safety. Similarly, measuring seasonality based on environmental and socioeconomic indicators may be more desirable than a dummy variable. Additionally, a longer panel would provide more robust evidence of the effect of seasonality in urban areas. Also, the effect of Covid-19, which occurred before the second round of data collection, may influence our outcome variables like dietary diversity and households’ attitudes towards food safety and health. Further studies are required to decouple the effects of seasonality and Covid-19 on the outcome variables.

## Conclusions

A sustainable urban food system is one of the most crucial priorities of a growing Ghanaian urban population because of its impact on food security and public health. This study set out to address the issue of food safety and HDDS in urban areas because they are important determinants of food security and public health. We answered whether household food safety and dietary diversity changed across seasons. The study also provided evidence of the presence of selected foodborne pathogens in selected purchased food commodities from an urban market.

Our results emphasise that in developing countries like Ghana, food safety is a challenge in major city food markets and is associated with weather seasonality. However, weather seasonality in cities does not significantly affect urban households’ HDDS. Households’ average HDDS is 7.3 and, therefore, cannot be considered food insecure. However, they purchase foods from markets where selected samples tested positive for high levels of aflatoxin B1 and other harmful foodborne microorganisms. Therefore, although there is food availability, the safety of food from traditional open-air markets is not assured. Therefore, urban food security can be improved by enhancing, in addition to food availability and accessibility, food safety along the food supply chain.

Ghana’s current food system will require a transformation to become sustainable. The domestic market food supply chain lacks adequate monitoring and enforcement of food safety standards. The National Food Safety Policy (NFSP) identified the high informality of the sector in Ghana as accounting for many activities not sufficiently regulated and the difficulty with product traceability [105]. Therefore, in the short term, Ghanaian local authorities such as sanitation and public health officers at the various markets should enforce laws (e.g. Public Health Act, 2012 (Act 851)) and regulations on food safety. The food safety surveillance and early warning system in the NFSP should be able to prevent the outbreak of diseases and detect and seize unsafe foods before they get to the markets. In the long run, technology, innovation, and logistics investment are required to upgrade traditional open-air markets. Although food availability is not a challenge for urban households, the focus should be on food safety and accessibility (affordability), especially for the urban poor.

### Supplementary Information

**Additional file 1:** **Supplementary Table 1.** Robustness of the results of the effect of seasonality on households’ incidence of diarrhoea/vomiting using alternative estimation methods. **Supplementary Table 2.** Robustness of the results of the effect of seasonality on HDDS using alternative estimation methods.Additional tables of the research.

## Data Availability

The data that support the findings of this study are available from the NOURICITY project, but restrictions apply to the availability of these data, so they are not publicly available. Data are, however, available from the authors upon reasonable request and with permission of the NOURICITY project.

## Abbreviations

CRE: Correlated Random Effect
EA: Enumeration Area
FAO: Food and agricultural Organisation
FDA: Food and Drugs Authority
GSS: Ghana Statistical Service
HDDS: Household Dietary Diversity Score
MoH: Ministry of Health
NFSP: National Food Safety Policy
NMIMR: Noguchi Memorial Institute for Medical Research
PPMLHDFE: Poisson pseudo-maximum likelihood estimator with multiple levels of fixed effects
WASH: Water, Sanitation and Hygiene
WHO: World Health organisation

## Footnotes

1 Corn dough is maize that is soaked in water for about 2 days, drained and milled into fine flour.
