# Supplementary material for: Food safety and dietary diversity in African urban cities: evidence from Ghana
Source: BMC Public Health. 2024 Mar 25;24:888. doi: 10.1186/s12889-024-18297-0 (PMC10962103; doi:10.1186/s12889-024-18297-0)
Supplement: Supplementary file 1 — Additional file 1: Supplementary Table 1. Robustness of the results of the effect of seasonality on households’ incidence of diarrhoea/vomiting using alternative estimation methods. Supplementary Table 2. Robustness of the results of the effect of seasonality on HDDS using alternative estimation methods.Additional tables of the research. [file 12889_2024_18297_MOESM1_ESM.docx]

Supplementary Table 1: Robustness of the results of the effect of seasonality on households’ incidence of diarrhoea/vomiting using alternative estimation methods

| Variables | 1 | 2 |
| --- | --- | --- |
| Season (Dry) | 4.561*** | 5.814*** |
|  | (1.727) | (2.112) |
| Characteristics of household |  |  |
| Age of household head | -0.058 |  |
|  | (0.074) |  |
| Sex of household head (male) | -0.379 |  |
|  | (1.216) |  |
| Household size | 0.155 | 0.123 |
|  | (0.282) | (0.162) |
| *Education of household head^*^* |  |  |
| Primary | -8.628*** |  |
|  | (2.452) |  |
| Secondary | -5.183** |  |
|  | (2.403) |  |
| Tertiary | -3.947 |  |
|  | (3.360) |  |
| *Household wealth status* |  |  |
| Lower-middle | 0.427 | 0.591 |
|  | (0.492) | (0.516) |
| Middle | 0.017 | 0.370 |
|  | (0.561) | (0.535) |
| Upper-middle | -0.301 | -0.456 |
|  | (0.638) | (0.585) |
| Upper | 0.999 | 0.192 |
|  | (0.798) | (0.750) |
| Household food safety knowledge | 0.016 | 0.014 |
|  | (0.014) | (0.018) |
| *Marital status* |  |  |
| Single | -2.308 |  |
|  | (2.238) |  |
| Monogamous | 0.175 |  |
|  | (1.498) |  |
| Polygamous | 7.868* |  |
|  | (4.123) |  |
| Price of maize^+^ | 22.551** | 30.229*** |
|  | (8.889) | (10.741) |
| Price of tomatoes^+^ | 0.387 | 0.474 |
|  | (0.336) | (0.453) |
| *Employment status* |  |  |
| Employment status of household head | 0.226 | 0.461 |
|  | (0.660) | (0.638) |
| Percent of household members employed | 0.018* | 0.027** |
|  | (0.011) | (0.012) |
| *Self-reported covid-19 effect* |  |  |
| Affected price of staple foods | 1.088** | 0.895* |
|  | (0.431) | (0.501) |
| Affected price of vegetables | -1.979*** | -2.108** |
|  | (0.638) | (0.866) |
| Constant | -3.546** | -61.883*** |
|  | (1.707) | (21.724) |
| Time varying averaged regressors | Yes | No |
| Number of observations | 1212 | 190 |
| Number of unique households | 606 |  |

Robust standard errors in parentheses. *** p<0.01, ** p<0.05, * p<0.1

Col. 1: CRE Poisson estimation; Col.2: PPMLHDFE estimation

* Reference base for educational level is “No formal education”

+Real price of maize and tomatoes are computed based on ESOKO-Ghana December and June price averages from 2013-2020.

Supplementary Table 2: Robustness of the results of the effect of seasonality on HDDS using alternative estimation methods

| Variables | 1 | 2 |
| --- | --- | --- |
|  | HDDS | HDDS |
| Season (Dry) | -0.055*** | -0.024 |
|  | (0.017) | (0.120) |
| Characteristics of household |  |  |
| Age of household head | 0.001 | 0.005 |
|  | (0.001) | (0.006) |
| Sex of household head (male) | -0.037 | 0.156* |
|  | (0.023) | (0.094) |
| Household size | 0.022*** | 0.036** |
|  | (0.005) | (0.017) |
| *Education of household head^*^* |  |  |
| Primary | 0.001 | 0.165 |
|  | (0.036) | (0.157) |
| Secondary | 0.020 | 0.158 |
|  | (0.024) | (0.153) |
| Tertiary | 0.058** | -0.176 |
|  | (0.029) | (0.291) |
| Household wealth index | 0.034*** | 0.035*** |
|  | (0.004) | (0.009) |
| Household food safety knowledge | 0.003*** | 0.003*** |
|  | (0.001) | (0.001) |
| *Marital status of household head* |  |  |
| Single | -0.054 | -0.598*** |
|  | (0.038) | (0.186) |
| Monogamous | 0.019 | -0.333*** |
|  | (0.027) | (0.082) |
| Polygamous | -0.009 | -0.554** |
|  | (0.044) | (0.271) |
| Price of maize^+^ | 0.095*** | 0.137 |
|  | (0.030) | (0.639) |
| Price of tomatoes^+^ | 0.008* | 0.018 |
|  | (0.005) | (0.023) |
| *Employment status* |  |  |
| Employment status of household head | 0.030 | -0.013 |
|  | (0.024) | (0.042) |
| Percent of household members employed | 0.000 | 0.001* |
|  | (0.000) | (0.001) |
| *Self-reported covid-19 effect* |  |  |
| Affected price of staple foods | -0.048** | -0.031 |
|  | (0.019) | (0.030) |
| Affected price of vegetables | 0.021 | 0.028 |
|  | (0.021) | (0.034) |
| Constant | 1.446*** | 1.467*** |
|  | (0.100) | (0.126) |
|  |  |  |
| Number of observations | 1,212 | 1,212 |
| Number of unique respondents |  | 606 |

Robust standard errors in parentheses. *** p<0.01, ** p<0.05, * p<0.1

Col.1: Poisson estimation (pooled data); Col. 2: CRE Poisson estimation

* Reference base for educational level is “No formal education”

+Real price of maize and tomatoes are computed based on ESOKO-Ghana December and June price averages from 2013-2020.
